# Linear Growth Trajectories, Catch-up Growth, and Its Predictors Among North Indian Small-for-Gestational Age Low Birthweight Infants: A Secondary Data Analysis

**DOI:** 10.3389/fnut.2022.827589

**Published:** 2022-05-24

**Authors:** Bireshwar Sinha, Tarun Shankar Choudhary, Nitika Nitika, Mohan Kumar, Sarmila Mazumder, Sunita Taneja, Nita Bhandari

**Affiliations:** ^1^Centre for Health Research and Development, Society for Applied Studies, New Delhi, India; ^2^DBT/Wellcome India Alliance, Hyderabad, India; ^3^Department of Global Public Health and Primary Care, University of Bergen, Bergen, Norway; ^4^Knowledge Integration and Transformation Platform at Centre for Health Research and Development, Society for Applied Studies, New Delhi, India

**Keywords:** catch-up growth (CUG), linear growth, small for gestational age (SGA), low birthweight (LBW) infant, growth faltering

## Abstract

**Background:**

Low birthweight small-for-gestational-age (SGA-LBW) (birthweight below the 10th percentile for gestational age; SGA-LBW) infants are at an increased risk of poor postnatal growth outcomes. Linear growth trajectories of SGA-LBW infants are less studied in South Asian settings including India.

**Objectives:**

To describe the linear growth trajectories of the SGA-LBW infants compared with appropriate-for-gestational-age LBW (AGA-LBW) infants during the first 6 months of life. In addition, we estimated catch-up growth (ΔLAZ > 0.67) in SGA-LBW infants and their performance against the WHO linear growth velocity cut-offs. Additionally, we studied factors associated with poor catch-up growth in SGA-LBW infants.

**Methods:**

The data utilized came from an individually randomized controlled trial that included low birthweight (LBW) infants weighing 1,500–2,250 g at birth. A total of 8,360 LBW infants were included. For comparison between SGA-LBW and AGA-LBW infants, we presented unadjusted and adjusted estimates for mean differences (MDs) or risk ratios (*RR*s) for the outcomes of length, linear growth velocity, length for age *z*-score (LAZ) score, and stunting. We estimated the proportion of catch-up growth. Generalized linear models of the Poisson family with log links were used to identify factors associated with poor catch-up growth in SGA-LBW infants.

**Results:**

Low birthweight small-for-gestational-age infants had a higher risk of stunting, lower attained length, and a lower LAZ score throughout the first 6 months of life compared with AGA-LBW infants, with differences being maximum at 28 days and minimum at 6 months of age. The linear growth velocity in SGA-LBW infants compared with AGA-LBW infants was significantly lower during the birth–28 day period [MD −0.19, 95% confidence interval (*CI*): −0.28 to −0.10] and higher during the 3- to 6-month period (MD 0.17, 95% *CI*: 0.06–0.28). Among the SGA-LBW infants, 55% showed catch-up growth for length at 6 months of age. Lower wealth quintiles, high birth order, home birth, male child, term delivery, non-exclusive breastfeeding, and pneumonia were associated with the higher risk of poor catch-up in linear growth among SGA-LBW infants.

**Conclusion:**

Small for gestational age (SGA) status at birth, independent of gestational age, is a determinant of poor postnatal linear growth. Promotion of institutional deliveries, exclusive breastfeeding, and prevention and early treatment of pneumonia may be helpful to improve linear growth in SGA-LBW infants during early infancy.

**Clinical Trial Registration:**

[https://clinicaltrials.gov/], identifier [NCT02653534].

## Introduction

Small for gestational age (SGA) infants are at higher risk of mortality, poor postnatal growth, morbidities, and long-term neurodevelopmental outcomes compared with appropriate for gestational age (AGA) infants (1–4). Estimates from 2012 suggest that 62.5% of the SGA births globally are from South or Southeast Asia. In India, 36.1% [95% confidence interval (*CI*): 25–52.8%] of all live births are SGA, as per the INTERGROWTH 21st standards (5). Evidence suggests that SGA infants account for approximately 40% of the stunting among under-two children in the Indian population (6). However, the postnatal linear growth patterns of SGA infants have been less studied in South Asian settings, including India.

Previous studies have used different definitions and country-specific standards to define SGA births and catch-up growth, which makes it difficult to compare across studies (7, 8). The Brighton Collaboration Working Group has defined SGA infants as those with birthweight below the 10th percentile for gestational age, and for computing birthweight centiles, the INTERGROWTH-21st standards are globally accepted (5, 7). However, for measurement of catch-up growth, multiple cut-offs have been used including a change in length for age *z*-score (LAZ) of >0.67 between two-time points, or achieving an LAZ of >2 SD or >1.3 SD, or growth above the third percentile for LAZ of the WHO 2006 growth standards at any time during follow-up (8–11). A systematic review by Campisi et al. in 2019 reported that ∼70–80% of the SGA infants showed catch-up growth in the first year after birth and by 2 years of age, >85% had catch-up growth (8). However, the studies included in the review were from developed countries and had used inconsistent definitions for catch-up growth. Moving forward to enable future international comparisons, Campisi et al. (8) have suggested using the criteria of “>0.67 change in LAZ scores as per the WHO 2006 child growth standards” to define catch-up growth in SGA infants over a specified time period as this represents a clinically significant response.

In this analysis, using data from an intervention cohort of 8,402 low birthweight (LBW) infants in North India followed up from birth to 6 months of age, our first objective was to describe the linear growth trajectories of the low birthweight small-for-gestational-age (SGA-LBW) infants compared with appropriate-for-gestational-age LBW (AGA-LBW) infants. Second, to estimate the performance of SGA-LBW infants against different growth indicators, such as catch-up growth in length and the WHO linear growth velocity cut-offs at 3 and 6 months of age. Third, to study the factors associated with poor catch-up growth in SGA-LBW infants at 6 months of age.

## Materials and Methods

### Study Design and Population

The present study was a secondary analysis of data from an individually randomized, controlled trial conducted between 2015 and 2018 to assess the efficacy of promoting community-initiated kangaroo mother care (ciKMC) on post-enrollment neonatal and 6-month mortality in 8,402 LBW weighing between 1,500 and 2,250 g within 3 days of birth. The study was conducted in Faridabad and Palwal districts of Haryana, India. The ciKMC intervention included promotion and support of skin-to-skin contact and exclusive breastfeeding through home visits on days 1–3, 5, 7, 10, 14, 21, and 28 of life (12). All infants in the intervention and control arms received usual care, i.e., home-based postnatal care visits (on days 3, 7, 14, 21, 28, and 42) as implemented through the health system (13). The ciKMC intervention had a substantial effect on infant 6-month mortality but had no substantial effect on linear growth at 6 months of age (12). The trial was registered with ClinicalTrials.gov, NCT02653534.

### Procedures

Pregnant women were identified by a screening and enrollment team and followed-up regularly and with increasing frequency as the expected date of delivery approached. Newborn infants weighing between 1,500 and 2,250 g were enrolled within 72 h of birth if kangaroo mother care (KMC) was not initiated in the facility and written informed consent was obtained from the mothers or primary caregivers (12). We excluded infants who were unable to feed, had difficulty in breathing, had less than normal movements, had gross congenital malformations, KMC was initiated the in hospital, or whose caregivers intended to move away over the next 6 months or refused participation. For the anthropometric assessments and obtaining other clinical information, home visits were conducted at the age of 28, 90, and 180 days.

All anthropometric assessments were taken two times by a pair of workers using the standard techniques (14). Standardization exercises were conducted prior to study initiation and repeated every 6 months (15). An infantometer (model 417; Seca, Chino, CA, United States; sensitivity 0.1 cm) and a digital hanging weighing scale (AWS-SR-20; American Weigh Scales, Cumming, GA, United States; 10 g sensitivity) were used to assess length and weight, respectively (15). Standard weights and length measurement rods were used to calibrate the weighing scales and infantometers at regular intervals, respectively (15). Detailed methods of the primary trial are previously published (12, 16).

### Assessment of Gestational Age, Small for Gestation Age, and Appropriate for Gestational Age Status

Gestational age at the time of delivery was estimated from antenatal ultrasound reports in 5,372 (64.3%) women. If the ultrasound was not available, we estimated gestational age based on the last menstrual period as documented in hospital records or as per maternal recall, in the given order of preference. For defining SGA/AGA, we calculated birthweight centiles using “growth standards” package based on INTERGROWTH-21 standards in R software (17). Infants with birthweight below the 10th percentile for their gestational age were classified as SGA, and those with ≥10th percentile as AGA.

### Study Outcomes

The study outcomes for linear growth in the first 6 months of life were mean length, linear growth velocity, LAZ, and stunting rates. Linear growth velocity was defined as the change in length in centimeters with respect to previous time points, i.e., birth to 28, 28–90, 90–180, and birth to 180 days. LAZ according to the WHO 2006 standards were generated using “zscore06” package in Stata 16.0, TX, United States (18, 19) and scores <−6 were excluded (14). Stunting was defined as LAZ score <−2.

Poor catch-up in linear growth was defined as ΔLAZ ≤ 0.67 between two-time points, as per the WHO 2006 child growth standards (8, 20). Catch-up growth was estimated in the SGA infants for the periods of birth to 3 months and birth to 6 months.

### Statistical Analysis

Analyses were done using STATA16.0 (Stata Corp., College Station, TX, United States) and statistical software R version 3.3.3 (The R Foundation for Statistical Computing, Vienna, Austria). To compare linear growth trajectories across the groups of LBW infants, i.e., SGA and AGA, we estimated the mean and standard deviation (SD) of absolute length in centimeters, LAZ scores, and proportion stunted at birth, 28, 90, and 180 days of age. In addition, we estimated the mean (SD) of linear growth velocity for time periods of birth to 28, 28–90, 90–180, and birth to 180 days. To compare the two groups, i.e., SGA-LBW and AGA-LBW infants, we estimated unadjusted mean difference (MD) for continuous variables, e.g., length, LAZ scores, and risk ratio (RR) for binary variables, e.g., stunting. We presented both unadjusted estimates, and estimates adjusted for gestational age, intervention using generalized linear models of the Poisson family with log links. In a sensitivity analysis, we compared the linear growth trajectories between SGA and AGA within the subgroup of infants born preterm <37 weeks gestation.

We estimated the proportion of SGA-LBW infants showing catch-up growth >0.67 SD during the birth to 3-month and birth to 6-month periods. In addition, using the WHO linear growth velocity standards (21), we estimated the proportion of SGA-LBW infants who were above the median, −1 SD, and −2 SD cut-offs during birth to 3-month, and birth to 6-month periods.

To identify the predictors of poor catch-up growth at 180 days, we conducted univariable and multivariable regression analyses using generalized linear models of the Poisson family with log link. In the regression model, we included covariates that have been previously shown to be associated with growth (22–26). The covariates with *p* < 0.1 in the univariable analysis or those biologically plausible were used in the multivariable model (27). In the multivariable analysis, we adjusted for the intervention, i.e., ciKMC and accounted for clustering within households. We reported both unadjusted and adjusted *RR* and its 95% *CI*s of poor catch-up growth for each predictor variable. We have reported *Akaike’s information criteria* (AIC) and *Bayesian information criteria* (BIC) value and have calculated the receiver operating characteristic area under curve (ROC-AUC) as a part of regression model diagnostics.

## Results

**FIGURE 1:**
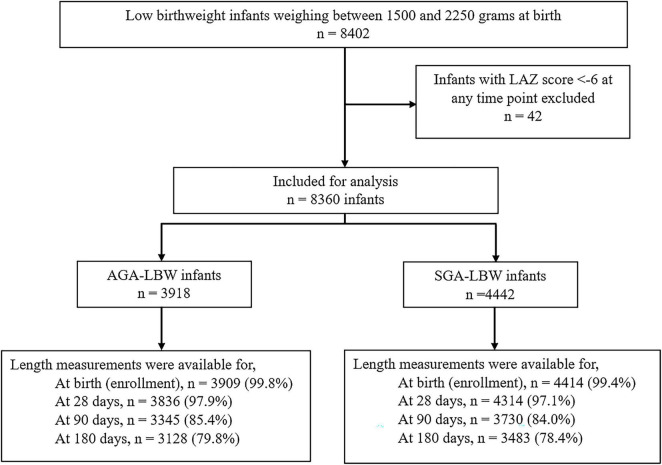
Flowchart showing the number of infants with length measurements included in the analysis.

Of the total 8,402 LBW infants, 42 infants with an LAZ score <−6 were excluded from the analysis. In the 8,360 LBW infants included, 3,918 were AGA and 4,442 were SGA. In the included infants, length measurements were available for almost all at birth, 97% at 28 days, 85% at 90 days, and 79% at 180 days of age (Figure 1). The mean (SD) gestational age of the AGA-LBW infants was 34.2 (1.6) weeks and that of SGA-LBW infants was 37.1 (1.2) weeks. Overall, 64.2% (5,369/8,360) were preterm; all AGA-LBW infants were preterm, and among SGA-LBW infants 32.6% (1,451/4,442) were preterm. The proportions of SGA and AGA infants exclusively breastfed at 3 months were 45.8 and 43.0%, respectively.

### Growth Trajectory

In the SGA-LBW and AGA-LBW infants, the mean (SD) length at birth was 44.7 (1.6) cm and 44.5 (1.5) cm, and that at 180 days of age was 62.4 (2.4) cm and 62.5 (2.4) cm, respectively. The mean difference (SD) in length between SGA-LBW and AGA-LBW infants adjusted for gestational age was the highest at 28 days (MD −0.98, 95% *CI*: −1.09 to −0.88) and the lowest at 180 days of age (MD −0.73, 95% *CI*: −0.89 to −0.58, Table 1).

**TABLE 1:** Linear growth patterns in small for gestational age (SGA) and appropriate for gestational age (AGA) low birthweight infants at different time points.

Characteristics	Time-point	SGA-LBW	AGA-LBW	Unadjusted mean difference/IRR	Adjusted^b^ mean difference/RR
Attained length, cm: mean (SD)	Birth^a^	44.67 (1.57)	44.54 (1.53)	0.12(0.06−0.19)	−0.82(−0.91to−0.72)
	28 days	49.40 (1.85)	49.22 (1.86)	0.18(0.10−0.26)	−0.98(−1.09to−0.88)
	90 days	56.20 (2.11)	56.11 (2.12)	0.08(−0.02to0.18)	−0.89(−1.02to−0.75)
	180 days	62.38 (2.35)	62.49 (2.35)	−0.11(−0.22to0.01)	−0.73(−0.89to−0.58)
Linear growth velocity, cm: mean (SD)	Birth^a^-28 days	4.72 (1.45)	4.66 (1.46)	0.05(−0.01to0.12)	−0.19(−0.28to−0.10)
	28-90 days	6.81 (1.45)	6.92 (1.46)	−0.11(−0.18to−0.04)	0.06(−0.04to0.15)
	90-180 days	6.23 (1.54)	6.37 (1.61)	−0.14(−0.22to−0.06)	0.17(0.06−0.28)
	Birth-180 days	17.75 (2.19)	17.95 (2.23)	−0.20(−0.31to−0.10)	0.07(−0.08to0.22)
LAZ score: mean (SD)	Birth*^a^*	−2.66(0.84)	−2.73(0.82)	0.06(0.03−0.10)	−0.46(−0.51to−0.41)
	28 days	−2.40(0.96)	−2.49(0.97)	0.09(0.05−0.14)	−0.53(−0.59to−0.48)
	90 days	−2.06(1.02)	−2.11(1.02)	0.04(−0.01to0.09)	−0.47(−0.53to−0.40)
	180 days	−1.89(1.04)	−1.85(1.05)	−0.05(−0.10to0.01)	−0.38(−0.45to−0.31)
Stunting^c^: n/N (%)	28 days	2,734/4,314(63.6)	2,596/3,836(67.7)	0.94(0.89−0.99)	1.24(1.16−1.34)
	90 days	1,846/3,730(49.4)	1,737/3,345(51.7)	0.95(0.89−1.02)	1.37(1.25−1.50)
	180 days	1,552/3,483(44.7)	1,367/3,128(43.9)	1.02(0.95−1.10)	1.33(1.20−1.47)

*^a^Birth measurements were within 3 days of birth. ^b^Adjusted for gestational age and intervention. ^c^For stunting, i.e., length for age z-score (LAZ) < −2 SD, risk ratio (RR) is estimated.*

The 6-month linear growth velocity during the period from birth to 180 days in SGA-LBW and AGA-LBW was 17.8 (2.2) and 17.9 (2.2) cm. Compared with AGA-LBW infants, the linear growth velocity in SGA-LBW infants was substantially lower in the neonatal period (adjusted MD −0.19, 95% *CI*: −0.28 to −0.10), and was significantly higher during the 90- to 180-day period (adjusted MD 0.17, 95% *CI*: 0.06–0.28). The difference in linear growth velocity during other time periods was not statistically significant.

At birth, the LAZ score of SGA-LBW and AGA-LBW infants was −2.7 (0.8) and −2.7 (0.8), respectively. At 180 days of age, the LAZ scores in SGA-LBW and AGA-LBW infants were −1.9 (1.0) and −1.9 (1.1), respectively. Adjusted analysis showed that the LAZ score of SGA-LBW infants was substantially lower than AGA-LBW infants at all time points of measurement (Table 1). The adjusted MD in LAZ score between SGA-LBW and AGA-LBW infants was maximum at 28 days and (MD −0.53, 95% *CI*: −0.59 to −0.48) and minimum at 180 days of age (MD −0.38, 95% *CI*: −0.45 to −0.31).

The proportion of stunting in SGA-LBW and AGA-LBW infants at 28 days was 64 and 68%, and that at 180 days was 45 and 44%, respectively. Adjusted analysis showed that the risk of stunting was higher by 24, 37, and 33% in SGA-LBW infants against AGA-LBW infants at 28, 90, and 180 days of age, respectively (Table 1).

The findings were similar in the subgroup of preterm infants (Supplementary Table 1).

### Catch-up Growth in Length Among Low Birthweight Small-for-Gestational-Age Infants

**FIGURE 2:**
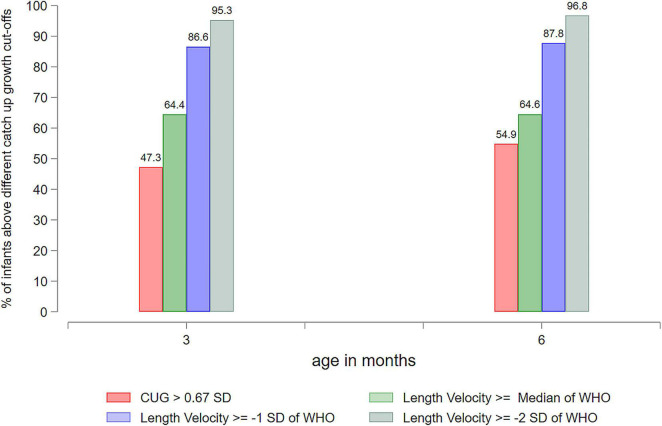
Performance of low birthweight small-for-gestational-age (SGA-LBW) infants with respect to different growth cut-offs at 3 and 6 months of age.

Among the SGA-LBW infants, 47% (1,760/3,724) showed catch-up growth by 3 months, and 55% (1,908/3,477) showed catch-up growth by 6 months. In AGA-LBW infants, 59% (1,890/3,226) were above the cut-off for catch-up growth at 6 months. As per the WHO length velocity standards from birth to 6 months, 65, 88, and 97% of the SGA-LBW infants were above the cut-offs for median, −1 SD and −2 SD, respectively (Figure 2). Among the AGA-LBW infants, 68, 88, and 97% were above the median, −1 SD, and −2 SD of the WHO length velocity standards from birth to 6 months, respectively.

### Predictors of Poor Catch-up in Linear Growth at 6 Months Among Low Birthweight Small-for-Gestational-Age Infants

**FIGURE 3:**
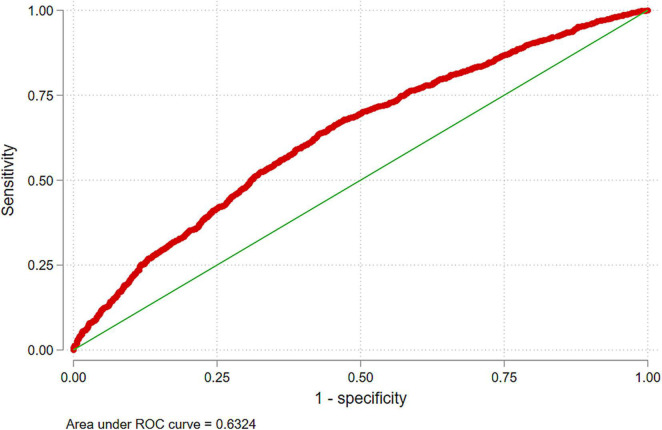
A receiver operating characteristic (ROC) curve of the multivariable model to predict poor catch-up growth in SGA-LBW infants.

Multivariable analysis showed that the adjusted risk of poor catch-up growth was substantially lower in the infants born to the least poor (*RR* 0.74, 95% *CI*: 0.64–0.86; Table 2) or less poor families (*RR* 0.86, 95% *CI*: 0.76–0.97) compared with the poorest families. Infants born at hospitals had a lower risk of poor catch-up growth against those born at home (*RR* 0.88, 95% *CI*: 0.81–0.96). Higher birth order infants (>4) had a greater risk of poor catch-up growth compared with infants born to primiparous mothers (*RR* 1.26, 95% *CI*: 1.09–1.47). The *RR* of poor catch-up growth was 0.89 (95% *CI*: 0.83–0.96) in the girl child vs. boys. Exclusive breastfeeding at 3 months was associated with a lower risk of poor catch-up growth (*RR* 0.89, 95% *CI*: 0.83–0.96). Preterm infants (<37 weeks gestational age) had a lower risk of poor catch-up growth (*RR* 0.82, 95% *CI*: 0.75, 0.89) than term infants. History obtained (H/O) pneumonia was associated with a higher risk (*RR* 1.24, 95% *CI*: 1.07, 1.44) of poor catch-up growth. We did not find any substantial association between diarrhea, hospitalization, or the study intervention, i.e., ciKMC with poor catch-up growth. The ROC-AUC of our multivariable regression model to identify predictors of poor catch-up growth was 63.2% (Figure 3).

**TABLE 2:** Factors associated with poor catch-up in linear growth status at 6 months among low birthweight small-for-gestational-age (SGA-LBW) infants.

Variables	Unadjusted RR (95% *CI*)	Adjusted^1,4^ RR (95% *CI*)	*P*-value (adjusted)
***Sociodemographic factors***			
**Maternal age, year**			
≤20	Reference	Reference	
21–29	1.03 (0.95, 1.13)	0.98 (0.88, 1.08)	0.644
≥30	1.31 (1.15, 1.50)	1.04 (0.89, 1.22)	0.609
**Maternal education**			
Educated	Reference	Reference	
Not educated	1.30 (1.21, 1.39)	1.06 (0.97, 1.15)	0.191
**Wealth quintile**			
Poorest	Reference	Reference	
Very poor	0.91 (0.82, 0.99)	0.95 (0.86, 1.06)	0.361
Poor	0.84 (0.76, 0.93)	0.94 (0.84, 1.05)	0.254
Less poor	0.75 (0.67, 0.84)	0.86 (0.76, 0.97)	0.018
Least poor	0.60 (0.53, 0.68)	0.74 (0.64, 0.86)	0.000
**Social category with reservations^2^**			
SC/ST	Reference	Reference	
OBC	1.05 (0.98, 1.14)	1.04 (0.96, 1.12)	0.337
Unreserved	0.71 (0.63, 0.79)	0.82 (0.73, 0.93)	0.002
***Birth-related factors***			
**Birth order**			
1	Reference	Reference	
2–4	1.22 (1.13, 1.32)	1.15 (1.05, 1.27)	0.002
>4	1.53 (1.36, 1.72)	1.26 (1.09, 1.47)	0.002
**Place of birth**			
Home	Reference	Reference	
Hospital	0.78 (0.72, 0.84)	0.88 (0.81, 0.96)	0.006
***Infant factors***			
**Sex**			
Male	Reference	Reference	
Female	0.90 (0.84, 0.97)	0.89 (0.83, 0.96)	0.001
**Gestational age category**			
Term ≥37 weeks	Reference	Reference	
Preterm <37 weeks	0.84 (0.78, 0.92)	0.82 (0.75, 0.89)	0.000
**Exclusively breastfed at 3 months**			
No	Reference	Reference	
Yes	0.90 (0.84, 0.97)	0.89 (0.83, 0.96)	0.004
**Hospitalization at 6 months**			
No	Reference	Reference	
Yes	1.12 (0.98, 1.26)	1.06 (0.94, 1.20)	0.329
**H/O diarrhea^3^**			
No	Reference	Reference	
Yes	1.04 (0.96, 1.13)	1.03 (0.95, 1.11)	0.485
**H/O pneumonia^3^**			
No	Reference	Reference	
Yes	1.35 (1.16, 1.57)	1.24 (1.07, 1.43)	0.004
**Study group**			
Control	Reference	Reference	
Intervention	1.00 (0.93, 1.08)	1.01 (0.94, 1.09)	0.684

*^1^Adjusted estimates are based on multivariable regression (predictor model). We have also adjusted for the intervention and accounted for any household clustering.*

*^2^OBC (other backward class)—term used by the GOI to classify socially and educationally disadvantaged category of population, SC/ST (schedule caste/schedule tribe)—historically disadvantaged indigenous people as identified by the Government of India.*

*^3^Based on history obtained (H/O) at 3 and 6 months visits (for last 14 days).*

*^4^Akaike’s information criteria (AIC) = 1.60, Bayesian information criteria (BIC) = −25705.1.*

## Discussion

**FIGURE 4:**
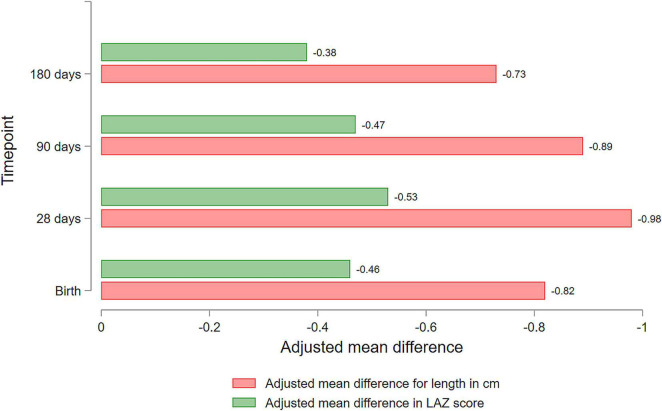
Mean difference (MD) in attained length and length for age *z*-scores (LAZ) between appropriate-for-gestational-age LBW (AGA-LBW) and SGA-LBW infants.

The findings showed that SGA-LBW infants in the study population had a higher risk of stunting, lower attained length, and LAZ score throughout the first 6 months of life compared with AGA-LBW infants, with the differences being maximum at 28 days and minimum at 6 months of age (Figure 4). The linear growth velocity in SGA-LBW infants compared with the AGA-LBW infants was lower during the neonatal period but was substantially higher during the 3- to 6-month period. More than half of the SGA-LBW infants met the criteria of catch-up growth for length at 6 months of age. Poor catch-up in linear growth among SGA-LBW infants was associated with lower wealth quintiles, high birth order, home birth, male child, term delivery, non-exclusive breastfeeding, and pneumonia.

Previous studies have observed the linear growth patterns in SGA and/or LBW infants but reports on the longitudinal growth of SGA-LBW infants is limited in Indian settings. In an earlier study using data from the third national health and nutritional examination survey in the United States (1988–1994), it was shown that despite catch-up, the SGA infants remain shorter and lighter compared with AGA or LGA infants from 2 to 47 months of age (28).

A recent study in Australia suggested that catch-up growth (defined by ≥0.67 SD change) is more frequent in SGA infants compared with AGA infants with normal intrauterine growth and most of the catch-up growth is observed at approximately 4 months of age (29). A systematic review in 2019 (8) that included 11 studies with full-term SGA infants showed that 87% of the children achieved catch-up growth across all the different definitions used, at latest follow-up (ranging between 1 and 18 years). The review reported that 58% of the SGA infants achieve catch-up growth at 6 months age, while 69–82% infants showed catch-up by 1 year of age. The most common definitions used to classify SGA births were a birthweight of <–2 SD, followed by a birthweight of less than the 10th percentile. To define catch-up growth, the most common definitions used were HAZ of >−2 SD or ΔLAZ > 0.67. A study in Western India that followed-up 247 LBW infants with 73% being SGA, reported that 80% of the infants were above the −2 SD HAZ score at 4 years of age.

The observation from our study corroborates with the previous reports and suggests that more than half of the SGA-LBW infants in our population in North India demonstrate catch-up growth during the first 6 months of life. However, SGA-LBW infants continue to remain relatively shorter than the AGA-LBW infants adjusted for gestational age at 6 months of age. In a longitudinal survey of full-term SGA babies followed-up to 1 year of age, belonging to upper socio-economic strata and representing North-Western India, SGA infants had significantly lower weight and length than AGA infants (30). Our study, along with previous reports, suggests that SGA status at birth, independent of gestational age, is a determinant with postnatal growth trajectory (28). The accelerated linear growth in early infancy in the SGA-LBW infants seems to compensate for intrauterine growth restriction, and the SGA infants not showing catch-up growth may be a high-risk group for short stature in adult life (31–33).

The association of poor catch-up in linear growth with lower wealth quintiles, high birth order, non-exclusive breastfeeding, and pneumonia, found in our study was similar to observations in previous studies (23, 34). We found that SGA-LBW infants born at term are at a higher risk of poor catch-up growth compared with preterm infants. This may be explained by the faster compensatory postnatal growth rate in preterm infants as also reported in previous studies (35). The observed lower risk of poor catch-up growth in girls compared with the boys corroborates to the fact that boys are born shorter than girls relative to the gender-specific international norms and continue to remain below these norms during the first 1,000 days (34, 36). The observed reduced risk of poor catch-up growth in hospital-born infants may be explained by better healthcare seeking. The findings highlight the importance of improving modifiable factors, such as institutional deliveries, exclusive breastfeeding, and prevention or early treatment of pneumonia to promote better linear growth of SGA-LBW infants in the first 6 months of life.

Beyond estimating catch-up growth by the definition of >0.67 SD change, we used the different cut-points of the WHO linear growth velocity standards to demonstrate the substantial variability in the proportions when different definitions are used (Figure 2). Currently, the definition of catch-up growth lacks clear consensus. The findings underscore the critical need to have standard definitions for catch-up growth in infants to enable comparison across studies and settings (8).

Our study is one of the largest studies on LBW infants reported to date in the Indian population with rigorous longitudinal measurements of anthropometry till 6 months of age. However, there are some limitations. First, ultrasound-based gestational age was not available in 36% of the women. However, we checked the proportions of catch-up growth and its determinants (results not shown) among the infants where ultrasonography (USG)-based gestational age was available, and the results were similar. Second, additional information on the maternal and paternal height, and fetal growth restriction could have been valuable, which has known association with postnatal growth. Lastly, as rapid catch-up growth in early life has implications on future cardio-metabolic risks (37), longer follow-up with measures of body composition could be helpful. In future, it may be worthwhile to plan longitudinal follow-up cohorts where serial measurement of fetal growth as well as postnatal growth is captured up to 2 years of life to be able to study and compare growth patterns of different subgroups of infants, such as LBW, SGA, preterm, as well as normal term-AGA infants parallelly.

## Data Availability Statement

The raw data supporting the conclusions of the article can be made available by the authors on request, without undue reservations.

## Ethics Statement

The study involved human participants. It was reviewed and approved by Ethics Review Committee, Centre for Health Research and Development, Society for Applied Studies, New Delhi; the Regional Committee for Medical and Health Research Ethics in Norway; and World Health Organization, Geneva. Written informed consent to participate in this study was provided by the mothers/primary caregivers of the infants included.

## Author Contributions

BS: conceptualization, data acquisition, data analysis and interpretation, writing the first draft, manuscript editing, and finalization. TC: conceptualization, data analysis and interpretation, manuscript editing, and finalization. NN: data analysis and writing the first draft of the manuscript. MK: manuscript editing and finalization. SM, ST, and NB: investigators of the original study-obtained funding, data access, and critical revision of the manuscript for important intellectual content. All authors read and approved the final manuscript.

## Conflict of Interest

The authors declare that the research was conducted in the absence of any commercial or financial relationships that could be construed as a potential conflict of interest.

## Publisher’s Note

All claims expressed in this article are solely those of the authors and do not necessarily represent those of their affiliated organizations, or those of the publisher, the editors and the reviewers. Any product that may be evaluated in this article, or claim that may be made by its manufacturer, is not guaranteed or endorsed by the publisher.

## Abbreviations

AGA: appropriate for gestational age
CUG: catch-up growth
GLMM: generalized linear mixed-effects model
IQ: intelligence quotient
KMC: kangaroo mother care
LAZ: length for age *z*-score
LMIC: lower- and middle-income countries
SD: standard deviation
SGA: small for gestational age.
