# Supplementary material for: Linear Growth Trajectories, Catch-up Growth, and Its Predictors Among North Indian Small-for-Gestational Age Low Birthweight Infants: A Secondary Data Analysis
Source: Front Nutr. 2022 May 24;9:827589. doi: 10.3389/fnut.2022.827589 (PMC9173003; doi:10.3389/fnut.2022.827589)
Supplement: Supplementary file 1 [file Data_Sheet_1.PDF]

Linear growth trajectories, catch up growth and its predictors among North Indian small-for-gestational age low birthweight infants: a secondary data analysis

Bireshwar Sinha, Tarun Shankar Choudhary, Nitika, Mohan Kumar, Sarmila Mazumder, Sunita Taneja, and Nita Bhandari

Supplementary table 1. Linear growth patterns in SGA and AGA low birthweight preterm infants at different time points

Characteristics	Time-point	SGA-LBW	AGA-LBW	Unadjusted Mean diff/ RR	Adjusted[b] Mean diff/RR
Attained Length, cm: Mean (SD)	Birth[a]	44.00 (1.58)	44.55 (1.53)	-0.55 (-0.64 to -0.46)	-1.02 (-1.12 to -0.92)
	28 d	48.63 (1.95)	49.22 (1.86)	-0.59 (-0.71 to -0.47)	-1.21 (-1.33 to -1.09)
	90 d	55.53 (2.21)	56.11 (2.12)	-0.59 (-0.73 to -0.45)	-1.09 (-1.24 to -0.94)
	180 d	61.99 (2.39)	62.49 (2.35)	-0.50 (-0.66 to -0.34)	-0.86 (-1.03 to -0.69)
Linear growth velocity, cm: Mean (SD)	Birth[a]-28 d	4.61 (1.52)	4.66 (1.46)	-0.05 (-0.14 to 0.04)	-0.20 (-0.30 to -0.10)
	28-90 d	6.88 (1.57)	6.92 (1.46)	-0.04 (-0.14 to 0.06)	0.06 (-0.04 to 0.17)
	90-180 d	6.49 (1.58)	6.37 (1.61)	0.12 (0.01 to 0.23)	0.24 (0.12 to 0.36)
	Birth-180 d	18.00 (2.20)	17.95 (2.23)	0.05 (-0.10 to 0.20)	0.14 (-0.03 to 0.30)
LAZ score: Mean (SD)	Birth[a]	-3.05 (0.83)	-2.73 (0.82)	-0.32 (-0.37 to -0.27)	-0.58 (-0.63 to -0.53)
	28 d	-2.83 (1.00)	-2.49 (0.97)	-0.34 (-0.40 to -0.28)	-0.66 (-0.72 to -0.60)
	90 d	-2.43 (1.06)	-2.11 (1.01)	-0.33 (-0.40 to -0.26)	-0.59 (-0.66 to -0.51)
	180 d	-2.13 (1.07)	-1.85 (1.05)	-0.29 (-0.36 to -0.21)	-0.47 (-0.54 to -0.39)
Stunting[c]: n/N (%)	28 d	1107/1399 (79.1)	2596/3836 (67.7)	1.17 (1.09 to 1.25)	1.33 (1.23 to 1.44)
	90 d	782/1230 (63.6)	1737/3345 (51.9)	1.22 (1.13 to 1.33)	1.46 (1.33 to 1.60)
	180 d	628/1164 (53.9)	1367/3128 (43.7)	1.23 (1.12 to 1.36)	1.41 (1.27 to 1.57)

[a]Birth measurements were within three days of birth
[b]Adjusted for gestational age and intervention
[c]For stunting i.e. LAZ<-2SD, Risk Ratio (RR) is estimated
